# Auditory functional magnetic resonance imaging in dogs – normalization and group analysis and the processing of pitch in the canine auditory pathways

**DOI:** 10.1186/s12917-016-0660-5

**Published:** 2016-02-20

**Authors:** Jan-Peter Bach, Matthias Lüpke, Peter Dziallas, Patrick Wefstaedt, Stefan Uppenkamp, Hermann Seifert, Ingo Nolte

**Affiliations:** Klinik für Kleintiere, Stiftung Tierärztliche Hochschule Hannover, Bünteweg 9, 30559 Hannover, Germany; Fachgebiet für Allgemeine Radiologie und Medizinische Physik, Stiftung Tierärztliche Hochschule Hannover, Bischofsholer Damm 15, 30173 Hannover, Germany; Medizinische Physik, Universität Oldenburg, 26111 Oldenburg, Germany

**Keywords:** fMRI, Functional magnetic resonance imaging, Auditory system, Dog, Normalization, Pitch

## Abstract

**Background:**

Functional magnetic resonance imaging (fMRI) is an advanced and frequently used technique for studying brain functions in humans and increasingly so in animals. A key element of analyzing fMRI data is group analysis, for which valid spatial normalization is a prerequisite. In the current study we applied normalization and group analysis to a dataset from an auditory functional MRI experiment in anesthetized beagles. The stimulation paradigm used in the experiment was composed of simple Gaussian noise and regular interval sounds (RIS), which included a periodicity pitch as an additional sound feature. The results from the performed group analysis were compared with those from single animal analysis. In addition to this, the data were examined for brain regions showing an increased activation associated with the perception of pitch.

**Results:**

With the group analysis, significant activations matching the position of the right superior olivary nucleus, lateral lemniscus and internal capsule were identified, which could not be detected in the single animal analysis. In addition, a large cluster of activated voxels in the auditory cortex was found. The contrast of the RIS condition (including pitch) with Gaussian noise (no pitch) showed a significant effect in a region matching the location of the left medial geniculate nucleus.

**Conclusion:**

By using group analysis additional activated areas along the canine auditory pathways could be identified in comparison to single animal analysis. It was possible to demonstrate a pitch-specific effect, indicating that group analysis is a suitable method for improving the results of auditory fMRI studies in dogs and extending our knowledge of canine neuroanatomy.

## Background

The auditory sense plays a significant role in many aspects of a dog’s life. It is important for the communication with humans and conspecifics and helps detect environmental threats such as motor vehicles [1]. An impairment of the hearing sense can severely impact a dog’s ability to function in its environment. Different methods have been used to examine the auditory function in dogs [2–5], among them electroaudiometry, which has also become the most important test for the clinical evaluation of hearing in canine patients [6]. Electroaudiometry is an objective, easy to perform and minimally invasive technique for evaluating the hearing function in dogs and other mammals [7]. Still, conventional electroaudiometry, which assesses the hearing function through electrodes placed subcutaneously around the skull, provides only very limited information about the spatial origin of the measured signals [8] and does not allow for an examination of the cortical auditory functions [9]. More detailed examinations of the central hearing functions with electrodiagnostic methods, as have been performed in cats, are feasible but highly invasive [10].

One of the most important techniques in hearing research in humans is functional magnetic resonance imaging (fMRI) [11]. fMRI is a noninvasive technique with a high spatial resolution capable of identifying brain regions reacting to specific stimuli presented to the subject during an fMRI-scanning-session. To do this, fMRI utilizes the BOLD (blood oxygenation level dependent) effect. The BOLD effect originates from an increase in metabolic activity and blood flow in activated parts of the central nervous system. As the increased blood flow exceeds the increased demand for oxygen, there is an increase in the ratio of oxygenated hemoglobin to deoxygenated hemoglobin in the activated areas, which can be detected as a slight signal rise in special fMRI sequences [12]. fMRI has been used in a huge number of studies to identify the regions responsible for the perception of different auditory features in the human brain [13–15], including the perception of pitch [16, 17]. As the auditory cortex is the location for performing higher auditory functions, these studies have highly improved our understanding of auditory perception in humans [11, 15] and other vertebrates [18, 19].

Due to the fact that it is required for the subject to remain motionless during the scanning process, it is in most cases necessary to employ anesthesia or physical restraint in fMRI experiments in animals [20–24]. Using physical restraint in awake animals is not only problematic due to ethical reasons, but in addition to this the results of such experiments may be influenced by stress or other neural activity induced by lying in the scanner. For these reasons, most fMRI experiments performed in animals use anesthesia to inhibit subject movement [20, 21, 24, 25].

Unfortunately, anesthesia strongly reduces the extent of the measured BOLD effect, making it difficult to detect neural responses via fMRI [26]. Even in awake humans, the detectable BOLD signal change is rather small and there are many factors influencing the intensity of the measured MRI signal. Therefore, it is necessary to obtain many images to distinguish signal changes elicited by stimuli from random signal changes: with sufficient numbers of repetitions, random signal changes tend to average out, while signal changes due to stimulation persist [27].

A technique frequently used in fMRI-studies in humans is normalization [28–30]. Normalization allows for the combined analysis of data across several subjects participating in a study, thereby increasing the number of images available and improving the study’s statistical power [31]. In addition to this, normalization reduces the influence of intersubject variability on the measured results and allows for the comparison of the neural activity of different groups (e. g. persons suffering from a certain disease compared to healthy individuals) [30]. For the present study, anatomical images and functional scans following auditory stimulation of ten anesthetized beagle dogs were acquired using MRI. One of two stimulation paradigms used was comprised of two different kinds of auditory stimuli: 1. simple Gaussian noise stimuli and 2. regular interval sounds (RIS) including a periodicity pitch as an additional sound feature [32]. First results of this study, including the findings of single animal analysis and the comparison of the two stimulation paradigms used, have already been published [33]. For the present article the functional data of each dog was normalized to a template image, which was developed by Datta et al. [34] to be used as a template for canine fMRI studies, and a statistical group analysis was performed. A statistical parametric map displaying the activated areas in the canine brain following auditory stimulation was generated and a region of interest (ROI)-analysis was performed. Afterwards, the results of the group analysis were compared to the results of single dog analysis. Finally, the effect of periodicity pitch on the activation in the ascending stages of the canine auditory system was examined by comparing the activation elicited by simple Gaussian noise to the activation produced by RIS. The results of these examinations were used to address the following questions: 1. Do normalization and group analysis improve the detection of activated regions along the auditory pathways in dogs? 2. Is the amount of incidental activation, which does not show any connection to the auditory pathways, reduced in the group analysis in comparison to the single animal analysis? 3. Are there regions which show an increased activation following stimulation with sounds including pitch in comparison to simple Gaussian noise?

## Results

**Fig. 1:**
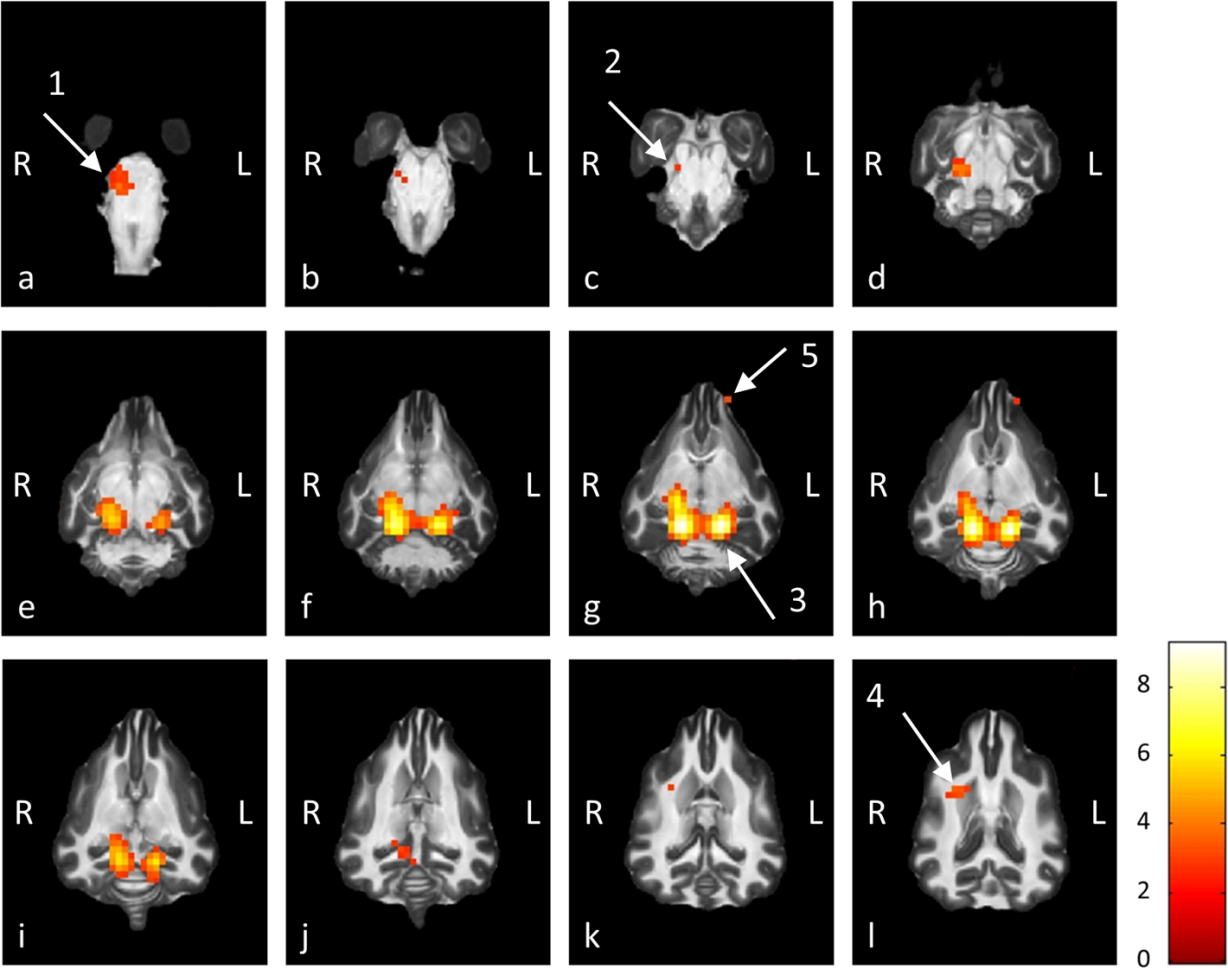
Group Analysis of auditory fMRI data following binaural stimulation with sparse temporal sampling paradigm. Functional data following auditory stimulation was collected for each dog using a T2* weighted functional MRI sequence. Afterwards the data was normalized to Datta’s atlas of the dog brain [34] and a statistical group analysis was performed. In the images above, all regions showing significant activation are displayed as colored pixels, which were superimposed onto dorsal slices of Datta’s brain atlas [34]. The gap between two adjacent slices is 2 mm. The colorbar indicates the t-values of the activated voxels. The pictures show unilateral activation of the right superior olivary nucleus in the brainstem (**a**-**b**, arrow 1). This activation can be traced dorsally along the lateral lemniscus (**c**-**d**, arrow 2) to the much larger bilateral activation of the caudal colliculi and further rostrally to the medial geniculate nuclei (**e**-**j**); merging activations marked with arrow 3. An activation matching the position of the right internal capsule can be found on pictures **k**-**l** (arrow 4). In addition to this, a small activated area was found in a region which is not normally associated with the auditory system (**g**-**h**, arrow 5). No significantly activated voxels were found in the auditory cortex

**Fig. 2:**
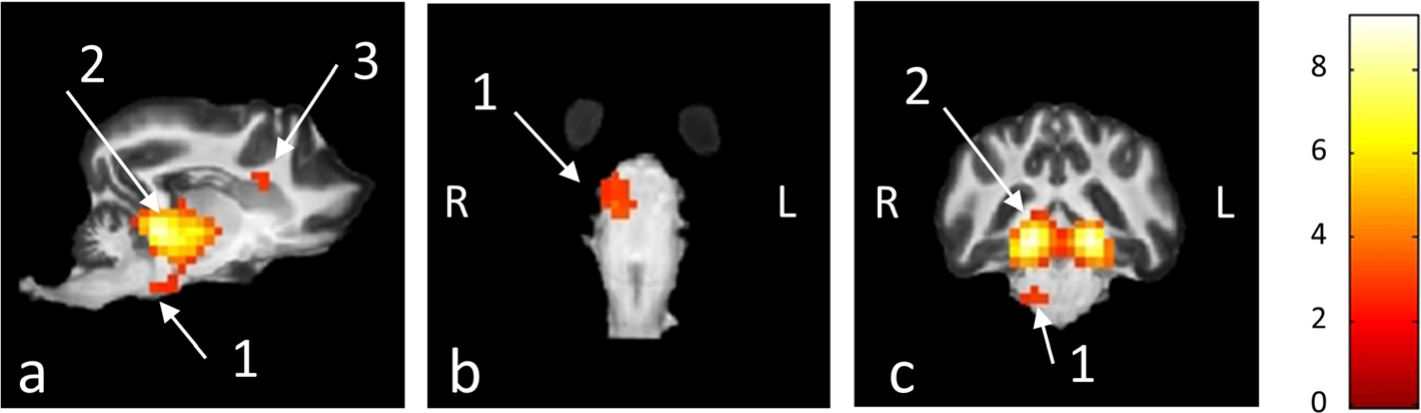
Unilateral activation of the olivary nucleus and the internal capsule. The image displays the unilateral activation of the right superior olivary nucleus (arrow 1), which was found following group analysis of the functional data obtained with sequence 1. The activation is displayed in sagittal (**a**), dorsal (**b**) and transverse (**c**) planes. In image (**a**), the activation can be traced along the lateral lemniscus to the much larger activation of the right caudal colliculus (arrow 2), which is also displayed on image (**c**). Image a also displays the dorsal aspect of the activation of the internal capsule already seen in Fig. 1 (k-l). It has to be noted that the part of the activation seen here (arrow 3) exceeds the anatomical boundaries of the internal capsule

Two of the ten dogs participating in the study were used to test the experimental setup and optimize the stimulation paradigms and functional imaging sequences, resulting in the inclusion of the functional data sets of eight dogs in the final study. A comparison of all sound conditions vs. silence yielded the following results: with sequence 1, which used the sparse temporal sampling method [35], significantly activated voxels were found in the regions of interest (ROIs) defined for the caudal colliculi (CC) and the medial geniculate nuclei (MGN) in the group analysis (Fig. 1). In comparison to the single animal analysis, where each ROI showed significantly activated voxels in every dog, no activation was evident in the ROI created for the temporal cortex (TC). In group analysis additional clusters of activated voxels were found matching the position of the right superior olivary nucleus, lateral lemniscus and internal capsule, which are known to have an auditory function in mammals (Figs. 1, 2) [14, 36, 37].

**Fig. 3:**
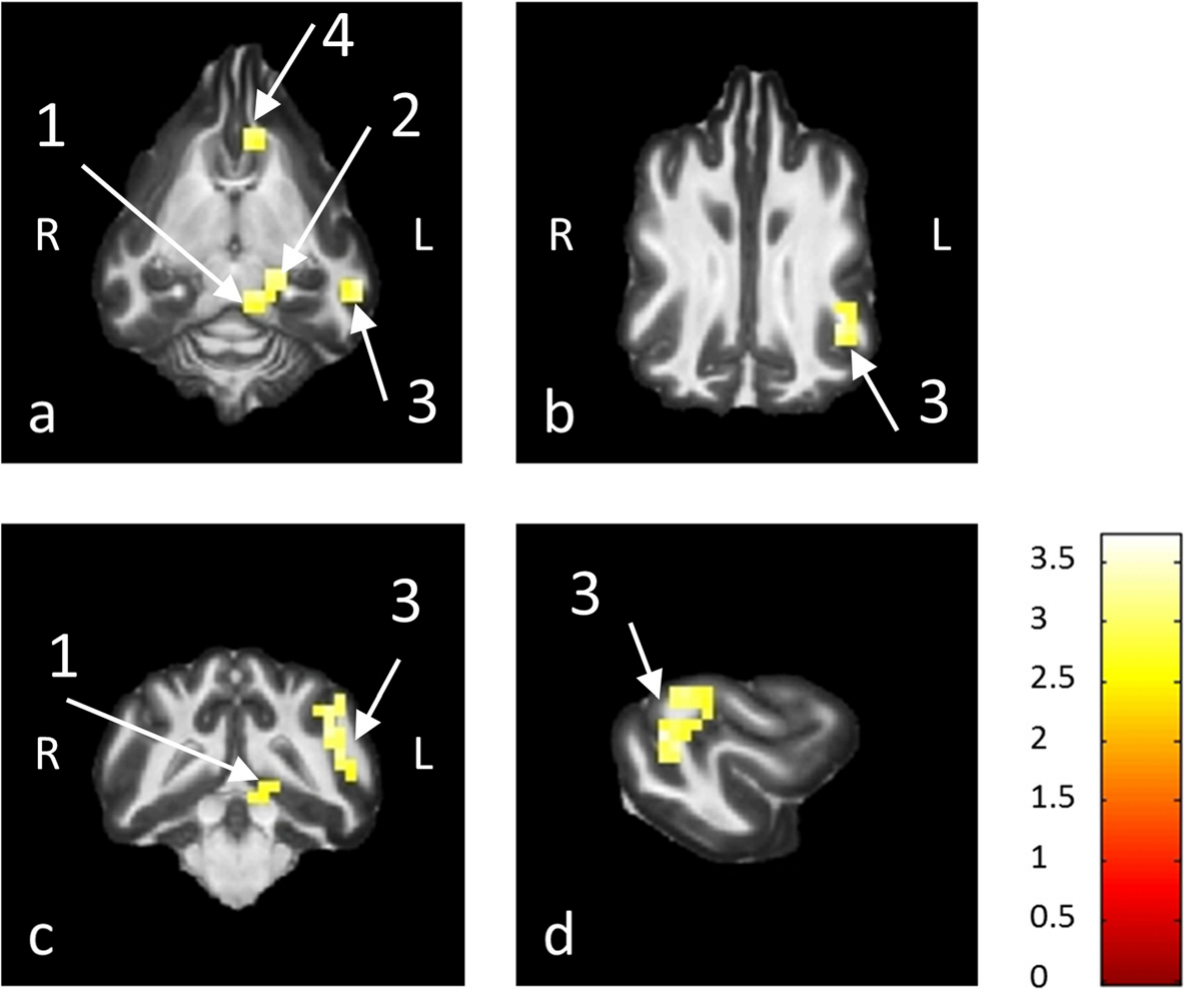
Group Analysis of auditory fMRI data following stimulation with continuous scanning paradigm. The group analysis of the functional data obtained with sequence 2 in combination with paradigm 2 (continuous stimulation) yielded unilateral activation of the left caudal colliculus (arrow 1), medial geniculate nucleus (arrow 2) and gyrus ectosylvius (arrow 3), which is part of the auditory cortex. These activations are displayed in dorsal (**a**, **b**), transverse (**c**) and sagittal (**d**) sections. In addition to these activations of the auditory pathways, an activated area is seen at the rostral aspect of the left caudate nucleus (**a**, arrow 4)

With sequence 2 significantly activated voxels were found in 4 dogs in the CC region, 3 dogs in the MGN region and 5 dogs in the TC region in the single animal analysis. The group analysis of the functional data acquired with sequence 2 showed significantly activated voxels in all ROIs, including a large cluster of activated voxels in the left temporal cortex. The activation in the temporal cortex was located in the ectosylvian gyrus (Fig. 3), which is consistent with lesion studies in dogs [38, 39] and other recent fMRI studies in dogs and cats [40, 41]. Though it was not possible to detect any cortical activation with the sparse temporal sampling paradigm, this paradigm elicited considerably higher activation in the subcortical auditory pathways. The statistical values calculated for the different ROIs can be found in Table 1*.* Following group analysis, t-values were considerably higher for all defined ROIs in comparison to the average t-values of the single animal analysis for the functional data obtained with sequence 2. For the data acquired with sequence 1, a higher t-value following group analysis was only calculated for the MGN region. The t-value for the CC region was slightly reduced in comparison to the single animal analysis. The t-value for the TC region, which was negative in the single animal analysis, was further reduced.

**Table 1:** Statistical values obtained with sequences 1 and 2 in the single animal and group analysis

Sequence 1						
	Single animal analysis (average)			Group analysis		
	Average t-value	Signal change (%)	Activated voxels (%)	t-value	Signal change (%)	Activated voxels (%)
CC	10.04	0.318	54.77	9.53	0.245	88.89
MGN	4.5	0.249	32.96	6.11	0.143	68.52
TC	-2.01	-0.032	0.52	-2.74	-0.045	0.08
Sequence 2						
CC	1.93	0.070	8.24	3.20	0.051	7.41
MGN	1.32	0.083	2.46	2.08	0.036	3.70
TC	0.77	0.023	2.05	3.20	0.050	3.88

The average t-value of all voxels included in the ROI, the mean percentage signal change between sound and silence conditions and the percentage of voxels showing significant activation are listed for the different sequences and ROIs (*CC* caudal colliculi, *MGN* medial geniculate nuclei, *TC* temporal cortex). The statistical values for the subcortical ROIs are higher with sequence 1, whereas only sequence 2 recorded a positive t-value and signal change for the cortical ROI

**Fig. 4:**
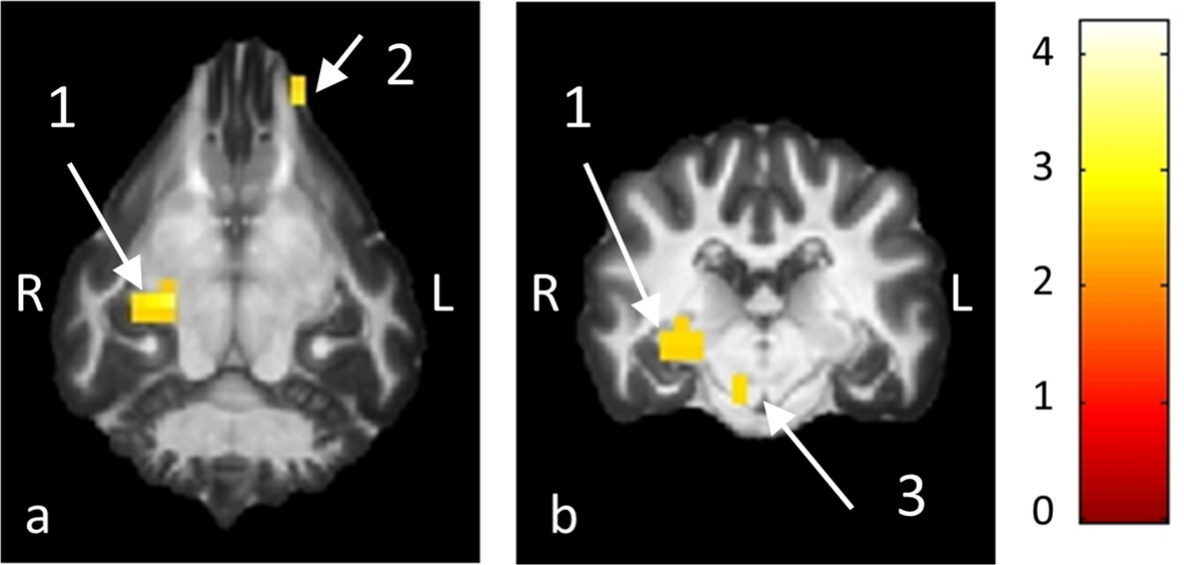
Comparison of RIS with simple Gaussian noise. The comparison of RIS with simple Gaussian noise using the functional data obtained with sequence 1 showed a significant contrast for the right medial geniculate nucleus (arrow 1), which is displayed in the dorsal (**a**) and the transverse (**b**) plane. In addition to this, small activations can be seen in the images without spatial relation to any known structures of the auditory system (arrows 2, 3)

On comparing regular interval sounds (RIS) with simple Gaussian noise a significant BOLD contrast was found in the MGN region (Fig. 4). The contrast yielded no significant activation for the CC and TC regions, and again little activation was detected outside the ROIs for this contrast. The peak t-values for this comparison were t = 2.02 (CC-region), t = 3.27 (MGN-region) and t = 2.33 (TC-region).

## Discussion

One of the aims of this study was to examine whether normalization and group analysis improve the detection of BOLD activation in the canine auditory pathway following auditory stimulation. For the subcortical ROIs, significantly activated voxels were found in all regions for both stimulation paradigms following group analysis. As the same is true for the majority of dogs in the single animal analysis, a beneficial effect of the group analysis is not clearly supported by this finding.

Concerning the existence of activated voxels in the TC ROI in the single animal analysis and the lack of these in the group analysis with sequence 1, it has to be noted that the activated voxels in the single animal analysis were few and not always located in areas commonly associated with the auditory system. Thus, it seems likely that part of the activations in the TC ROI, which was comparatively large in comparison to the small ROIs defined for the subcortical parts of the auditory pathways, may have been incidental in the single animal analysis. Due to the large extent of the TC ROI it is also possible, that this region included voxels which were not part of the auditory system, thus leading to rather small average t-values for this area in comparison to the other ROIs. The activation in the TC ROI, which was found in the group analysis following continuous stimulation however, was large and located in the ectosylvian gyrus, which was identified as part of the auditory pathway in other studies [40, 41].

In addition to the activated regions found in the single animal analysis, additional activated areas were detected with sequence 1 at the location of the internal capsule and in the brainstem matching the position of the right superior olivary nucleus [42]. Though an auditory function of these structures could be suspected due to studies in other mammals, this study is the first to confirm these assumptions in dogs via fMRI. Since it was not possible to detect a significant activation in these areas in single animal analysis, these results indicate that normalization and group analysis are suitable for facilitating the detection of activated voxels along the auditory pathway in canine fMRI-studies, especially concerning smaller signal changes, which might otherwise be missed due to the decreased BOLD contrast associated with anesthesia. The digital atlas of the dog brain developed by Datta et al. [34], though derived from the brains of mixed-breed dogs, seems to be adequate as a template for the normalization and group analysis of fMRI data acquired in studies in beagles and possibly other mesaticephalic dogs. Whether the same is true for brachycephalic and doliocephalic dogs has to be examined in future studies.

The reason why some of the activation found with sequence 1 and sequence 2 was only detected on one side, although binaural auditory stimulation was applied, remains unclear. A possible explanation could be that the stimulation applied to both ears was not perceived in the same manner. Though great effort was invested on placing the earphones in identical positions in both external ear canals and performing the stimulation at the exact same sound level, a displacement of the earphones (originally designed for use in human subjects) could have resulted in different stimulation of the ears. In addition to this, the ear protectors placed above the earphones may have been positioned slightly differently on both sides, resulting in a decreased activation following auditory stimulation due to background noise.

Very few activated voxels were found which were not located in or directly adjacent to known structures of the auditory pathway. In fMRI studies in awake people, high statistical thresholds and additional measures like family-wise-error correction are used to distinguish random signal changes from signal changes due to stimulation. As it is common to use lower statistical thresholds in animals, it is of great importance to minimize the false positive results by other means. Group analysis seems to be a suitable method for this purpose.

Another benefit of normalization and group analysis as performed for this study is the generation of a dataset which represents the neural activity found in a group of different subjects, thereby reducing the influence of individual abnormalities in functional neuroanatomy. The resulting dataset and the template used for normalization and group analysis are freely available, allowing other researchers to easily compare and combine the results of this study with their own findings in future studies.

Another important aspect of the current study was the investigation of the effect of pitch on the auditory perception of the dog. Pitch-specific neural activity has already been identified in humans in numerous studies [16, 32, 43, 44]. In this study, the neural activation in the MGN ROI was significantly increased for stimuli including pitch as a sound feature in comparison to simple Gaussian noise. No significant difference was detected in other parts of the auditory system. Pitch is an important feature of sound that plays an eminent role in daily communication of people: The pitch of speech can inform the listener about the age, gender and emotional state of the speaker [45, 46]. Although it is difficult to investigate whether animals perceive sound in the same manner as humans, many studies suggest that the perception of pitch plays an important role in the communication of animals as well [47–50]. Due to the many similarities in pitch perception among species, animal models may provide information about the neural processes connected with the auditory perception in different species, including humans [44]. As it is possible to train dogs to remain motionless in the MRI-scanner during fMRI-experiments, as has been shown in recent studies [40, 51, 52], the dog model seems to be well suited for the research of the auditory system and other neural processes in animals in the future.

In addition to answering the questions asked at the beginning of this study, group analysis also provides new information concerning the suitability of the two stimulation paradigms for their use in auditory fMRI studies in dogs: while paradigm 1 seemed to be superior to paradigm 2 in the single animal analysis due to stronger activation of the subcortical auditory pathways, the considerable activation of the auditory cortex following group analysis shows that a continuous stimulation may also offer benefits. For these reasons, a general superiority of sparse temporal sampling over continuous scanning methods as indicated in various auditory fMRI studies in humans and monkeys [25, 35, 53] cannot be stated for canine auditory fMRI. The finding of a significant activation in the auditory cortex with sequence 2 in comparison to sequence 1, where no cortical activation was found in this study, is in accordance with a study in cats, where continuous stimulation resulted in a larger extent of cortical activation than stimulation with a sparse paradigm [41]. Hall et al. [41] did not find a significant difference between continuous und sparse stimulation for the subcortical parts of the auditory system, though.

## Conclusions

Following group analysis it was possible to show a pitch-specific effect in the canine brain and detect additional activated areas along the canine auditory pathways in comparison to single animal analysis. These findings support the assumption that group analysis is a suitable method for improving the results of auditory fMRI studies in dogs. As to the comparison of the two stimulation paradigms it can be summarized that stronger activation of the subcortical auditory pathways was detected with the sparse temporal sampling paradigm, whereas significant activation in the temporal cortex could only be found following stimulation with the continuous paradigm. Altogether fMRI offers interesting opportunities for future research concerned with canine hearing disorders and neuroanatomy. Regarding possible clinical applications of auditory fMRI in animal patients, the need of anesthesia hinders a widespread use in clinical practice, as other testing procedures like electroaudiometry, though lacking the detailed spatial information given by fMRI, are easier to perform and produce more reliable results in anesthetized animal patients.

## Methods

The study was designed as a prospective, experimental study.

### Animals

Ten healthy beagles were included in the study. The mean age of the beagles was 3.7 years (+-2.3 SD) and they had an average body weight of 16.0 kg (+-2.6 SD). Prior to the examination in the fMRI scanner, a physical examination and a neurological examination were performed to rule out any hearing impairments or an increased anesthetic risk. In addition to this, an otoscopic examination and a BAER test were conducted during the same anesthesia in which the MRI examinations took place. Details of the BAER examination and the performed anesthesia can be found in [33]. All participants of the study were clinic-owned beagles from the Small Animal Clinic of the University of Veterinary Medicine Hannover. All procedures were approved by the Animal Welfare Officer of the University of Veterinary Medicine Hannover and the Lower Saxony State Office for Consumer Protection and Food Safety, Oldenburg, Germany (TV-No. 33.9-42502-05-12A223).

### Stimuli

The stimuli used to elicit a BOLD response in the beagles’ brains were simple random noise stimuli (Gaussian noise, i. e., the digital signal was made of normally distributed random numbers), bandpass-filtered between 250 Hz and 4 kHz, and regular interval sounds (RIS), generated from the random noise by an iterative delay-and-add procedure. This stimulus, also known as iterated rippled noise, is perceived as a noise-like stimulus with a certain tonal component caused by the spectral ripples introduced by the delay-and-add procedure (see e.g. [16]). The pitch of an acoustic signal is the perceptual correlate of its periodicity. The distance between the spectral ripples is the inverse of the delay time and determines the perceived pitch. As long as the delay for the RIS generation is 10 ms or more, the spectral ripples will not be resolved by the frequency analysis of the basilar membrane. The long-term spectral excitation pattern is therefore very similar for the random noise and the RIS stimuli. The pitch is related to time-interval processing in the auditory system rather than to spectral peaks [16].

Noise and RIS stimuli were chosen for this study rather than pure tones since the broadband excitation of the auditory system is known to evoke rather solid activation of auditory areas in the brain. In addition, an analysis of the contrast between random noise and RIS allows for an estimation to be made of the significance of time-interval processing in the canine auditory system, which has not been demonstrated before. The delay for the stimuli used throughout this study was between 20 ms and 10 ms, corresponding to a periodicity pitch between 50 and 100 Hz.

All sound stimuli were created in Matlab (The Mathworks Inc., Natick, MA, USA) and presented to the subjects binaurally at a sound level of 90 dB SPL using fMRI compatible headphones (Sensimetrics S14 insert headphones, Sensimetrics corp., Malden, USA) in combination with canine ear covers (Mutt Muffs, Safe and Sound Pets, Westminster, USA) for additional hearing protection. The stimuli were arranged alternatingly with periods of silence using two different paradigms: paradigm 1 consisted of Gaussian noise, regular interval sounds (RIS) and silence, each condition being repeated 40 times and presented in random order. Paradigm 2 involved eight periods of silence and eight periods of RIS, each period lasting 30 s. Each stimulation paradigm was combined with a suitable imaging sequence (see below).

### Imaging

Data acquisition was performed on a 3 Tesla Philips Achieva MRI scanner (Royal Philips, Amsterdam, the Netherlands) in combination with 11 cm diameter circular surface coils. Prior to the acquisition of the functional data, high-resolution anatomical images in the dorsal plane were obtained from each dog with a T1 weighted sequence with repetition time (TR) = 11 ms and echo time (TE) = 5.2 ms with a field of view (FOV) of 220 mm and 0.7 mm × 0.7 mm × 0.7 mm isotropic voxels.

**Fig. 5:**
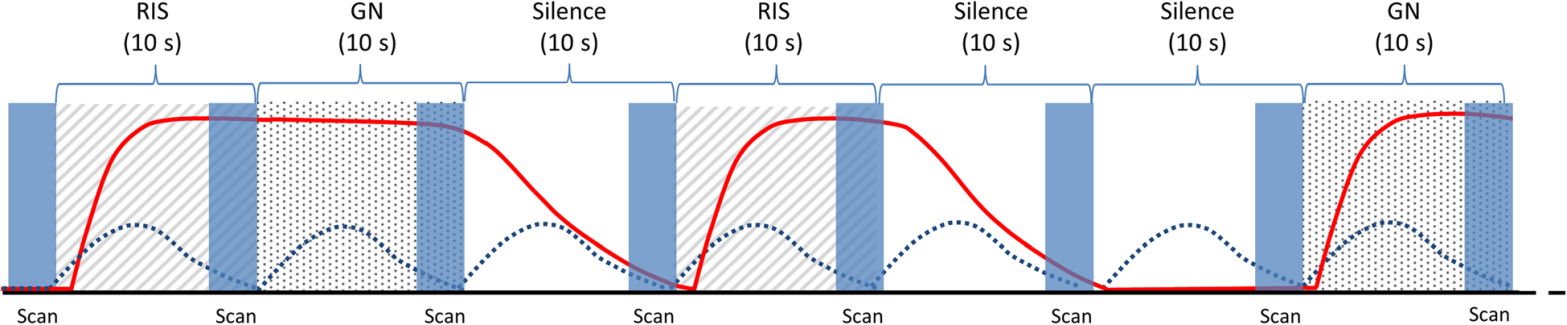
Design of the sparse temporal imaging paradigm. The sparse temporal imaging sequence initiates with an imaged brain volume (depicted in blue), which was not included into the final analysis to avoid T1 effects. Afterwards cycles of regular interval sound (RIS, hatched), simple Gaussian noise (GN, stippled) and silence (white) are presented to the dog in random order. By obtaining all scans of each volume in rapid succession at the end of each cycle, the sound stimuli can be presented in relative silence between the acquisitions of two volumes. Due to the delay in the hemodynamic BOLD response (red line), each scan measures the effect of the sound stimulation that occurred in the gap before the scan and is relatively uninfluenced by the response evoked by the scanner noise (dotted line)

Afterwards, two different echoplanar T2* weighted sequences with a slice thickness of 2 mm for 20 contiguous slices in the dorsal plane, a FOV of 192 mm, a matrix of 96 × 96 and an TE of 35 ms were used to acquire functional data sets. Sequence 1 used the sparse temporal sampling method [35], which is a well-established procedure for reducing the influence of the scanner noise on the obtained functional images in auditory fMRI-experiments. With this sequence, a volume of images was recorded every 10 s (TR = 10 s), all images of one volume were recorded within a period of 3 s, resulting in 7-s-gaps between the measurements in which stimuli could be presented unmasked by scanner noise (Fig. 5). With this sequence, 120 volumes were recorded in 20 min.

Sequence 2, which was combined with paradigm 2, was a conventional continuous sequence. With this sequence, 160 volumes were collected in 8 min (TR = 3000 ms, TE = 35 ms).

### Functional data analysis

MRI data were processed and analyzed using SPM (FIL, Welcome Trust Center for Neuroimaging, UCL, London, England) in combination with the ROI-analysis toolbox MarsBar [54]. The two functional datasets acquired in each dog were analyzed separately. First, all functional images were realigned to the first image of the series. Then, a mean functional image was generated for each dog, to which the anatomical image of the dog could be registered. For the single animal analysis, the following steps were performed without normalization of the functional data to a template image. Functional images were smoothed using a three-dimensional Gaussian filter (5 mm full width at half maximum). The general linear model was used with the standard hemodynamic response function provided by SPM as a reference to fit the measured time course of each voxel. Initially, all sound conditions combined were defined as the active condition in contrast to all periods in which no acoustic stimulus was presented as the rest condition. Afterwards, a *t*-test was used to test for differences between the two conditions. Following a recent auditory fMRI-study in anesthetized cats [21], the threshold for significant activation was set to *p* < 0.005 (no multiple comparisons) and a cluster size of at least 3 adjacent voxels. Singer’s ‘The Brain of the Dog in Section’ [42] and Pallazi’s ‘The Beagle Brain in Stereotaxic Coordinates’ [55] were used to assign the activated areas to the underlying anatomical structures.

ROIs were defined for three different anatomical regions, which are known to exhibit a detectable BOLD response in fMRI-studies in humans [14]: 1. the caudal colliculi (CC), 2. the medial geniculate nuclei (MGN) and 3. the temporal cortex (TC). Three statistical values were calculated for each ROI for both functional sequences used: 1. average t-value of all voxels included in the ROI, as a measure of significance of the difference found between active and rest conditions; 2. the mean percentage signal change between these two conditions; 3. the number of activated voxels at *p*-value 0.005 as a percentage of the total number of voxels included in the ROI. Afterwards, an average value across all subjects was calculated for each statistical value for each sequence and each ROI to be later compared to the values obtained in the group analysis. Details concerning the configuration and placement of the ROIs and the statistical values calculated for each ROI can be found in [33].

For group analysis, the realigned functional images were normalized to Datta’s brain atlas of the dog [34] before image smoothing. All subsequent steps of data preprocessing were performed similar to the single animal analysis. For statistical analysis, the data across all subjects were combined. Initially, the datasets acquired with the two functional sequences were analyzed separately. Afterwards, a combined analysis of the datasets was conducted. Like in the single subject analysis, all sound conditions were defined as the active condition in contrast to silence.

Finally, the influence of pitch on the elicited BOLD signal was examined by comparing the periods in which Gaussian noise stimuli (no pitch) were applied to periods with RIS-Stimulation (periodicity pitch). As the effect of periodicity pitch on the obtained BOLD signal was expected to be more feeble than the BOLD contrast in the comparison of sound vs. silence, the threshold for significant activation was reduced to *p* = 0.01 and no cluster size-threshold was applied. The peak t-values of the activated voxels in each ROI were determined for this comparison. As no Gaussian noise stimuli were included in paradigm 2, only data acquired with paradigm 1 in combination with imaging sequence 1 were used for this final step of data analysis.

### Data availability

The data sets supporting the results of this article are available in the Dryad repository: doi:10.5061/dryad.251h1.

## Abbreviations

BOLD: blood oxygenation level dependent
CC: caudal colliculi
fMRI: functional magnetic resonance imaging
FOV: field of view
MGN: medial geniculate nuclei
RIS: regular interval sounds
ROI: region of interest
TC: temporal cortex
TE: echo time
TR: repetition time
